# Analysis of Whole Transcriptome RNA-seq Data Reveals Many Alternative Splicing Events in Soybean Roots under Drought Stress Conditions

**DOI:** 10.3390/genes11121520

**Published:** 2020-12-19

**Authors:** Li Song, Zhenzhi Pan, Lin Chen, Yi Dai, Jinrong Wan, Heng Ye, Henry T. Nguyen, Guozheng Zhang, Huatao Chen

**Affiliations:** 1 Joint International Research Laboratory of Agriculture and Agri-Product Safety, Jiangsu Key Laboratory of Crop Genomics and Molecular Breeding, Co-Innovation Center for Modern Production Technology of Grain Crops, Yangzhou University, Yangzhou 225009, China; MZ120180906@yzu.edu.cn (Z.P.); albertmingliang@outlook.com (L.C.); daiyi@yzu.edu.cn (Y.D.); 2 National Center for Soybean Biotechnology and Division of Plant Sciences, University of Missouri, Columbia, MO 65211, USA; wanj@missouri.edu (J.W.); yehe@missouri.edu (H.Y.); nguyenhenry@missouri.edu (H.T.N.); 3 National Key Laboratory of Crop Genetics and Germplasm Enhancement, National Center for Soybean Improvement, Nanjing Agricultural University, Nanjing 210095, China; zgz@njau.edu.cn; 4 Institute of Industrial Crops, Jiangsu Academy of Agricultural Sciences, Nanjing 210014, China

**Keywords:** soybean, root, alternative splicing, drought response, transcriptome

## Abstract

Alternative splicing (AS) is a common post-transcriptional regulatory mechanism that modulates gene expression to increase proteome diversity. Increasing evidence indicates that AS plays an important role in regulating plant stress responses. However, the mechanism by which AS coordinates with transcriptional regulation to regulate drought responses in soybean remains poorly understood. In this study, we performed a genome-wide analysis of AS events in soybean (*Glycine max*) roots grown under various drought conditions using the high-throughput RNA-sequencing method, identifying 385, 989, 1429, and 465 AS events that were significantly differentially spliced under very mild drought stress, mild drought stress, severe drought stress, and recovery after severe drought conditions, respectively. Among them, alternative 3′ splice sites and skipped exons were the major types of AS. Overall, 2120 genes that experienced significant AS regulation were identified from these drought-treated root samples. Gene Ontology term analysis indicated that the AS regulation of binding activity has vital roles in the drought response of soybean root. Notably, the genes encoding splicing regulatory factors in the spliceosome pathway and mRNA surveillance pathway were enriched according to the Kyoto Encyclopedia of Genes and Genomes pathway enrichment analysis. Splicing regulatory factor-related genes in soybean root also responded to drought stress and were alternatively spliced under drought conditions. Taken together, our data suggest that drought-responsive AS acts as a direct or indirect mode to regulate drought response of soybean roots. With further in-depth research of the function and mechanism of AS in the process of abiotic stress, these results will provide a new strategy for enhancing stress tolerance of plants.

## 1. Introduction

Alternative splicing (AS) is an important posttranscriptional regulatory process for altering the structure of pre-mRNA that modulates the function, structure, and cell location of genes. AS has been found in many tissues and different development stages of eukaryotes and is essential in coordinating the growth and development of plants [1,2,3,4]. It has been reported that developmental stage-dependent AS events dramatically affect rice grain yield [5]. Additionally, many genes have been selected for increased AS complexity during the domestication of maize [6]. Therefore, AS has been assumed to have an important role in the developmental plasticity of plants. Additionally, the alternate protein variants derived from AS events may compete for interactions with the same factors and therefore function differently to cope with environmental changes [7]. 

With the advent of next-generation sequencing-based approaches, a large number of plant transcripts have been shown to be alternatively spliced under abiotic stress conditions in plants [8,9,10,11]. For example, the number of AS events increased significantly under cold or salt stress in *Arabidopsis* and cassava [12,13,14]. AS analysis in maize suggested that drought stress induced large developmental splicing changes in a tissue-dependent pattern [9]. AS regulation was shown to coordinate with transcriptional regulation to fine-tune the heat or heat/drought stress response in wheat [15]. In rice, the conserved AS of *dehydration responsive element binding protein 2B (DREB2B)* introduced a premature translation-termination codon (PTC) into the splicing isoform *OsDREB2B1* under non-stress conditions, which resulted in the production of a nonfunctional isoform. However, under high temperature or drought stress, the functional isomer OsDREB2B2 was significantly induced to improve the stress resistance of rice [16]. Wheat WDREB2, an orthologue of OsDREB2B, showed similar AS patterns under drought conditions, which highlighted the conservation of AS modulation during response to drought stress among different species [17,18]. Increased AS complexity in the rice maintainer HuHan2B also contributed to additional drought resistance during the breeding process [19]. Therefore, an in-depth analysis of the regulation mechanism of AS at the post-transcriptional level will help to understand the complex regulation of the plant response to environmental changes [20,21].

Soybean is a major crop that provides oil and protein for humans [22]. A recent study has shown that the majority of RNA splicing events occur in developing soybean embryos [23]. In soybean, more than 63% of multiexonic genes undergo AS [24]. There are five AS types in plants: intron retention (IR), skipped exon (SE), alternative 5′ splicing site (A5SS), alternative 3′ splicing site (A3SS), and mutually exclusive exon (MXE) [20]. IR is the dominant type of AS, even in response to abiotic stress [14,25,26,27,28]. 

Soybean is a drought-sensitive crop during the seedling stage, and drought stress is a major factor limiting global soybean growth and production [29]. The developmental plasticity of the soybean root system provides an opportunity to improve water and nutrient uptake and usage during environmental changes [30]. However, it is barely known how AS and transcriptional regulation participate in drought response in soybean root, although more recent research in plants provides a new perspective for AS as a promising regulator of root development. For example, the different patterns of AS impact root distribution and are related to the deep root system in rice [31]. Splicing factor 3b involves pre-mRNA splicing associated with root hair development in response to light signals in *Arabidopsis* [32]. In addition, different AS informs may respond differently to stress. The GATA gene family is involved in nitrate assimilation, and one of the members, *OsGATA23a*, showed high expression levels under salinity and drought conditions, whereas another alternative splice variant, *OsGATA23b*, did not respond to the above conditions [33]. Therefore, investigating the AS regulation and expression patterns of different AS isoforms under stress conditions may shed some light on the mechanism of abiotic stress response in soybean. Understanding the impact of AS on the plasticity of root development under abiotic stress conditions in soybean could also offer promising guidance to soybean breeders to develop drought-resistant cultivars [34]. 

In the present study, soybean (Williams 82 variety) roots under very mild, mild, and severe drought stress as well as water recovery after severe drought stress were used as materials in an RNA-sequencing (RNA-seq) experiment to identify the alternatively spliced isoforms and quantify the differential drought-responsive AS events. All five AS types were found in soybean roots in response to drought stress. The differentially spliced genes were determined and used to identify the enriched Gene Ontology (GO) terms and Kyoto Encyclopedia of Genes and Genomes (KEGG) pathways. Our results indicate that the skipped exon (SE) type was the most abundant AS event type. We also identified some novel AS events that might function in the drought response. These findings provide insight into AS regulation during soybean response to drought in the post-transcriptional process.

## 2. Materials and Methods 

### 2.1. Datasets and Treatments

The RNA-seq dataset (http://www.ncbi.nlm.nih.gov/sra/SRP067593, [35]) was used to test AS with replicate multivariate analysis of transcript splicing (rMATs) program in this study. Soybean variety Williams 82 (*G. max*) was used in this study. Four treatments—very mild stress (VMS), mild stress (MS), severe stress (SS), and water recovery after severe stress (SR)—were applied and monitored on the basis of the leaf water potential [35]. All plants were well-watered until V3 (three unfolded trifoliate leaves) stage before treatment. Each stress treatment corresponded to a well-watered control at the same growth stage. The VMS treatment involved 5 days of withholding water while other plants were well-watered as a corresponding control (VMS-WW). For MS treatment, water was withheld for 12 days while well-watered plants were used as corresponding control (MS-WW). For SS treatment, water was withheld for 19 days while other well-watered plants were used as a corresponding control (SS-WW). For SR treatment, plants were re-watered for 2 days after SS treatment while well-watered plants were used as a corresponding control (SR-WW). Therefore, the transcriptome datasets from a total of 24 libraries [(4 treatments + 4 controls) × 3 reps] were used in this study.

### 2.2. Reference-Based Transcriptome Structure Assembly

RNA isolation, library construction, and RNA sequencing data were consistent with the previous description in [35]. More mapping processes of RNA-seq reads were performed in this study to match the rMATs analysis. Except for fastx_toolkit_0.0.14 (http://hannonlab.cshl.edu/fastx_toolkit/), additional filtering for poor-quality bases was performed through SeqPrep (https://github.com/jstjohn/SeqPrep) and Sickle (https://github.com/najoshi/sickle). Furthermore, in order to identify novel splice sites with direct mapping to known transcripts and produce sensitive and accurate alignments, we used TopHat2 (http://ccb.jhu.edu/software/tophat/index.shtml, [36]) instead of TopHat for alignment with the *G. max* reference genome (*G. max* Wm82.a2.v1) and Phytozome v13 gene model in this study. The software StringTie (http://ccb.jhu.edu/software/stringtie/) was used to assemble the mapped reads, which were used to compare with the known transcripts in order to obtain novel transcripts. Novel transcripts were named as MSTRG plus numbers. 

### 2.3. AS Detection and Identification of Drought Stress-Responsive AS Events 

The software rMATs (version 4.0.1) [24] (http://rnaseq-mats.sourceforge.net/index.html) was used to identify AS events and analyze the differential AS events between samples. The junction count-only method was used in this study. We identified AS events with a false discovery rate (FDR) < 0.05 in comparison to AS events with a significant drought response. The classification of AS was as follows: SE: skipped exon, MXE: mutually exclusive exon, A5SS: alternative 5′ splice site, A3SS: alternative 3′ splice site, RI: retained intron. 

### 2.4. Differential Gene Expression Analysis

The differential expression analysis of RNA-seq data was performed between 2 different groups by using DESeq2 software (and by edgeR between 2 samples) [37,38]. The genes/transcripts with a false discovery rate (FDR) of <0.05 and absolute fold change ≥ 2 were considered as differentially expressed genes/transcripts. The expression values of the resulting genes or transcripts were presented as transcripts per million (TPM) values. 

### 2.5. Gene Ontology and Pathway Enrichment Analysis

All DSGs were mapped to GO terms in the Gene Ontology database (http://www.geneontology.org/) and gene numbers were calculated for every term. Significantly enriched GO terms in DSGs compared to the genome background were defined by hypergeometric test. The calculation of the *p*-value was performed with FDR correction, taking an FDR ≤ 0.05 as a threshold. The significantly overrepresented KEGG pathways were determined by Fisher’s exact test (*p*-value < 0.05) compared with the entire genome background. 

## 3. Results

### 3.1. Overview of the Sequencing Quality of RNA-seq Data for Identifying AS Events 

Twenty-four RNA-seq libraries were used to examine the regulation of AS in soybean roots under various drought stress conditions. These libraries included very mild drought stress (VMS), mild drought stress (MS), severe drought stress (SS), and recovery after severe drought (SR), with each stress treatment corresponding to a well-watered control at the same growth stage (WW). Each sample had three biological replicates and was sequenced separately [35]. Our previous study showed that thousands of genes were differentially expressed in soybean roots in response to various levels of drought stress [35]. Here, we used the same RNA-seq dataset to further examine the comprehensive profiles of AS events under those stress conditions. Instead of TopHat, TopHat2 was used in this study to identify novel splice sites with direct mapping to known transcripts, since the software can produce sensitive and accurate alignments, even for highly repetitive genomes or for those with pseudogenes [36]. In total, over 1.54 billion clean reads were utilized in downstream analysis for each sample, and the Q20 (base ratio > 20), Q30 (base ratio > 30), and GC content of the obtained clean data were calculated. The high percentage of Q30 (over 86.2%) in each sample suggested that a high accuracy of sequencing was achieved (Table S1). As shown in Figure S1A, the total filtered reads were uniformly distributed across all 20 soybean chromosomes. In addition, the coverage of sequencing results indicated that there was no significant bias in sequencing (Figure S1B). Therefore, the quality of assembly and sequencing data was high, which is consistent with previous reports [35], and it could be used for the detection and analysis of AS events. 

### 3.2. Identification of AS Events in Soybean Roots under Various Drought Treatments 

**Figure 1:**
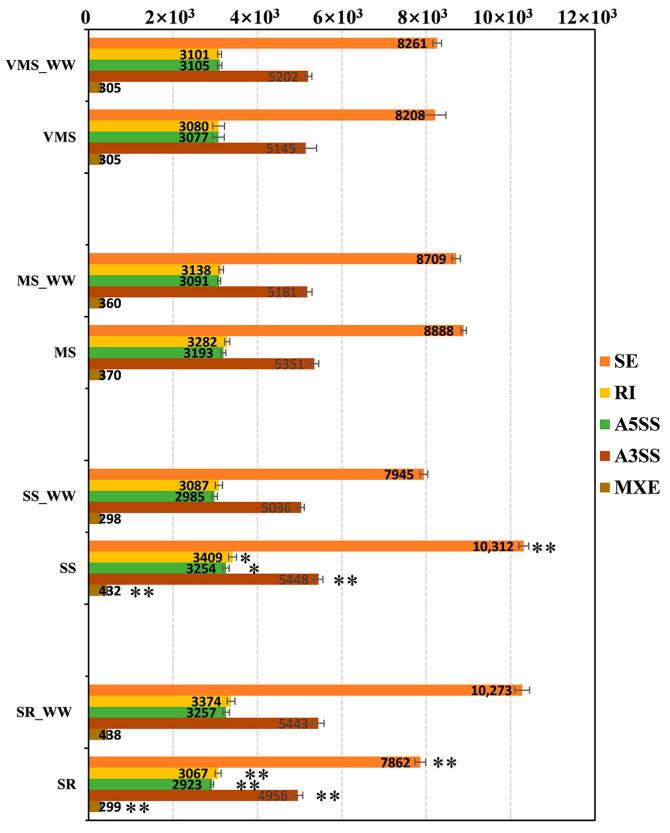
Categories and numbers of different types of alternative splicing (AS) events identified in the transcriptomes of soybean Williams 82 under various drought stress conditions. VMS: very mild drought stress, MS: mild drought stress, SS: severe drought stress, SR: water recovery after severe drought stress, SE: skipped exon, MXE: mutually exclusive exon, A5SS: alternative 5′ splice site, A3SS: alternative 3′ splice site, RI: retained intron. The data are expressed as mean ± standard deviation of three biological replicates. **, *p* < 0.01; *, *p* < 0.05.

High-quality reads were first mapped to the soybean reference genome (Williams 82, Ensembl version Glycine_max_v2.1) and then AS events were identified and quantified by the junction counts only method. All five AS types were identified in soybean roots with or without stress treatments. There was no significant difference in the number of these five AS types between well-watered control of very mild drought stress (VMS_WW) and VMS conditions or between well-watered control of mild drought stress (MS_WW) and MS conditions. However, the numbers of AS events increased significantly under the SS condition and decreased significantly under the SR condition (Figure 1). The differences in terms of AS numbers between replicates were smaller than that of treatments in SS- and SR-treated samples. Specifically, 10,312 SE-type AS events and 5448 A3SS-type AS events were detected under the SS condition, which was significantly greater than that of AS events under the SS_WW condition (*p* < 0.01). On the contrary, the number of AS events under the SR condition significantly decreased compared with that of the SR_WW and SS conditions. For example, only 7862 SE-type AS events were identified under the SR condition (Figure 1). In addition, the distributions of AS types under each condition were comparable—SE was the most abundant (41–45%) of the AS events out of all five AS types, followed by A3SS (24–26%); the number of A5SS and retained intron (RI) (14–16%) events were similar, but the number of MXE events (2%) was far smaller than that of the other AS types (Figure S2). These results suggest that the number of AS events increased significantly under the severe drought condition and SE was the major splicing type in soybean roots under various drought conditions. The number of splicing events increased with the deepening of the degree of drought while it decreased significantly after re-watering. 

### 3.3. Identification of Drought Stress-Responsive AS Events in Soybean Root 

**Figure 2:**
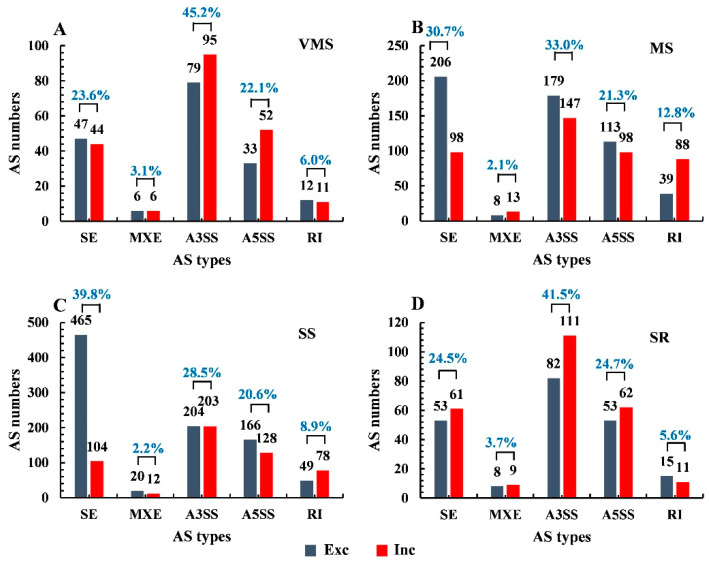
Comparison of the number and proportions of differential AS events under various drought conditions. (**A**) Under VMS condition, (**B**) under MS condition, (**C**) under SS condition, and (**D**) under SR condition. Exclusion is shown in blue, and inclusion is shown in red. The *y*-axis shows the number of AS events. The *x*-axis shows the types of AS.

The splicing isoforms of the genes that showed differential expression after stress treatments were called stress-responsive (drought) AS (DAS) events. This kind of event may be involved in regulating the plant response to drought stress. In this study, the “junction reads-based FDR (false discovery rate calculated from *p*-value) < 0.05” criteria were used to determine the DAS events. In total, 385, 989, 1429, and 465 stress-responsive DAS events were identified under VMS, MS, SS, and SR conditions, respectively. This result indicates that AS responses under the MS and SS treatments were enhanced compared with the VMS and SR treatments (Figure 2). Among all types of AS events, A3SS was the most abundant AS type under the VMS (45.2%), MS (33.0%), and SR (41.5%) conditions, but the number of SE-type (especially exclusion-type) events significantly increased under the MS and SS conditions (39.8%) compared to all the other AS types. In addition, the numbers of RI events under the MS and SS treatments were more than those of the VMS and SR treatments. Moreover, the number of alternative exon exclusions was more than the number of exon inclusions in A3SS and A5SS types under MS and SS conditions but was less than those of the VMS and SR conditions (Figure 2). These results suggest that the distribution of AS types and ratios of different isoforms under various drought conditions were different. 

**Figure 3:**
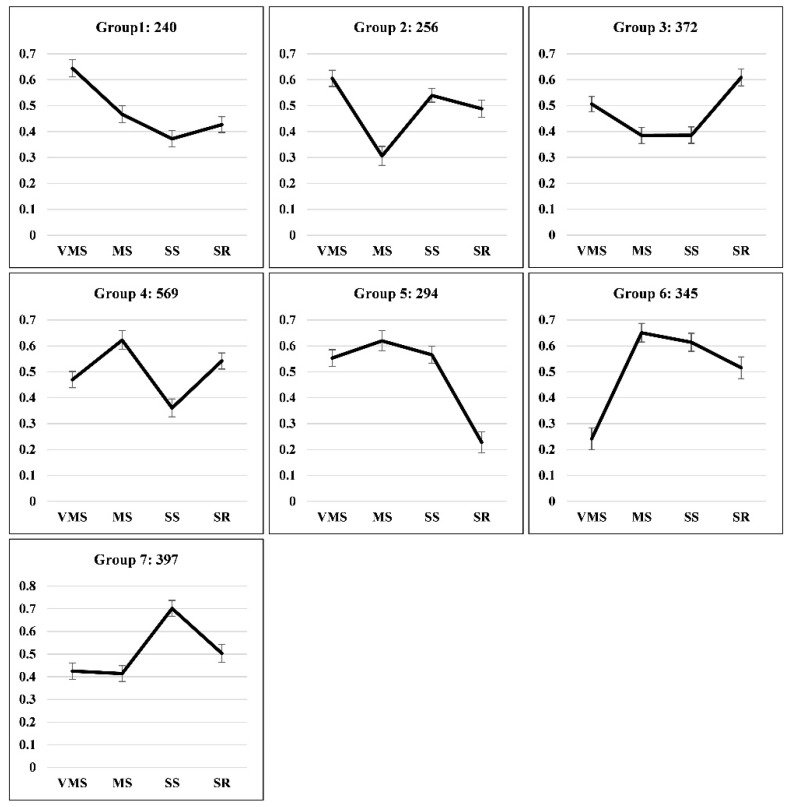
Cluster analysis of stress-responsive AS events in soybean root displayed on the basis of their isoform expression percentage (IEP) variations under various drought conditions. (IEP = Average ∆PSI (ww)/(Average ∆PSI (ww) + Average ∆PSI (stress)). The number of AS events in each cluster is listed. The *x*-axis shows various stress conditions, and the *y*-axis shows IEP value. The black lines indicate the trends of average IEP value for all AS events in each cluster.

The IEP (isoform expression percentage) changes have been used to classify the groups of stress-responsive AS events in wheat [15]. In the present study, the IEPs for all stress-responsive AS events were further clustered to reveal the magnitude of AS pattern changes (Figure 3). Seven groups were identified on the basis of AS drought-response profiles. Group 1, representing a total of 240 AS events, indicated responses to VMS treatment. Group 2 showed AS pattern changes under both VMS and MS treatments. Group 3 showed slight AS pattern changes under MS, SS, and SR treatments. There were 569 AS events in group 4, which included response to MS and SS treatments. Group 5 only showed significant changes of AS pattern under SR treatment, suggesting that SR treatment created a specific AS response. Groups 6 and 7 only exhibited significant changes of AS pattern in response to the VMS or SS treatments, respectively. Therefore, our present study revealed seven groups of AS events that responded differentially to different drought conditions.

### 3.4. Identification of Potential Drought Regulators via Analyzying Novel Drought-Responsive DSGs and DEGs 

**Figure 4:**
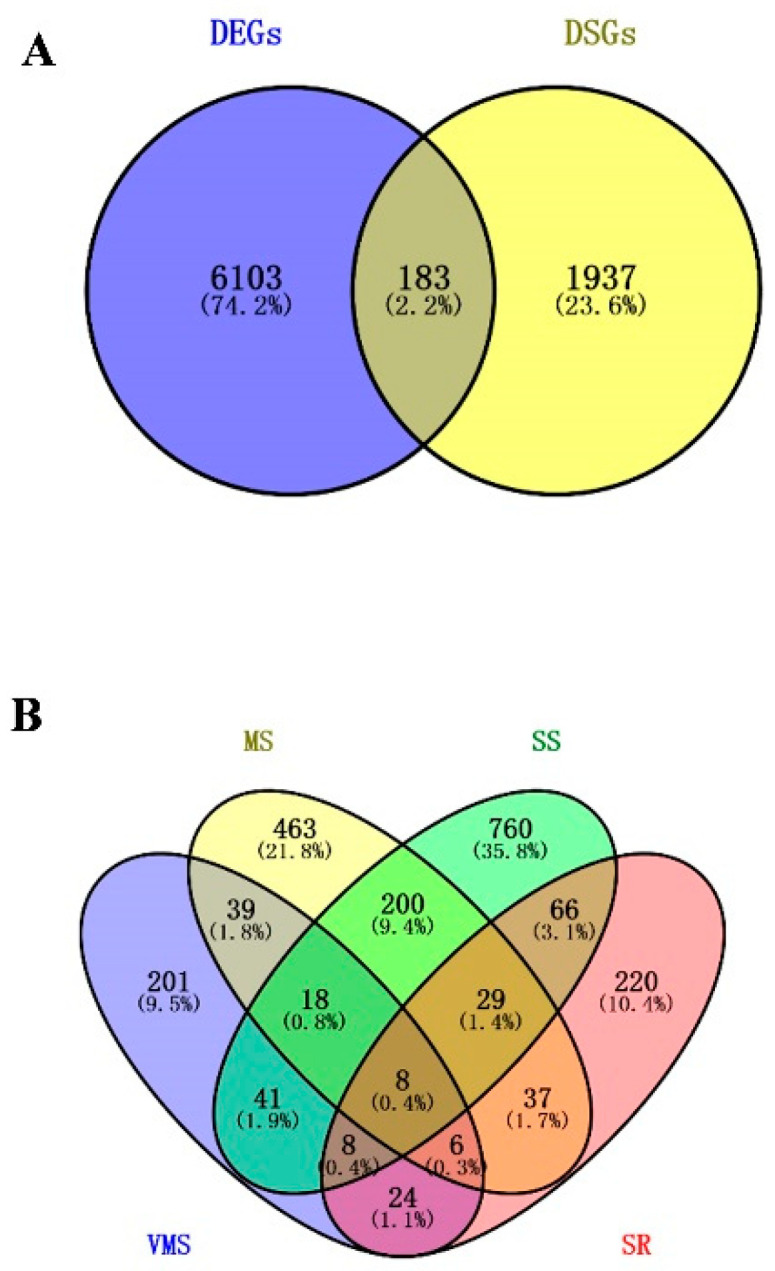
Venn diagram of differentially expressed genes (DEGs) and differentially spliced genes (DSGs). (**A**) Comparison analysis of overlapping between DEGs and DSGs under all drought conditions. (**B**) Comparison of DSGs under VMS, MS, SS, and SR conditions.

The genes with significantly differential drought stress-responsive AS events were named differentially spliced genes (DSGs). In this study, a total of 2120 DSGs (345, 801, 1130, and 398 under VMS, MS, SS, and SR treatments, respectively) were identified in soybean roots (Table S2). First, the DSGs and differentially expressed genes (DEGs) were compared and the results indicated that 183 genes showed significant drought-responsive expression patterns and AS regulation patterns (Figure 4A). Second, the DSGs were compared under various drought conditions, and the results showed that MS and SS shared the largest overlap—12% (255) of the DSGs overlapped between MS and SS treatments, while only 2.2–5.3% (46-111) of the DSGs overlapped between VMS/MS, SS/SR, VMS/SS, VMS/SR, or MS/SR (Figure 4B). Similarly, more stress-responsive AS gene overlap was found between MS and SS conditions (Figure S3). These results suggested that the mild drought-induced AS response was similar to those induced by severe drought stress, but distinct from VMS and SR. Notably, many genes encoding hormone-related proteins, drought-related proteins, splicing-related proteins, and heat shock-related proteins exhibited significant AS pattern changes (Figure S4). For example, drought-induced protein 19 exhibited significant AS pattern changes in response to the VMS, SS, and SR treatments (Figure S4A), and auxin efflux carrier family protein (Glyma.19G128800) underwent different AS events under different drought stresses (Figure S4D,E). These results indicate that many pathways enriched in DEGs were also found in DSGs.

To further investigate whether any novel AS events were identified and involved in soybean drought response in this study, we studied three types of AS (MXE, RI, and SE) events that significantly responded to drought stress (Table S3). First, more novel AS events were identified under the MS and SS conditions than under the VMS and SR conditions. Second, a total of 232 novel SE–type AS events were identified under all treatments (Table S3), suggesting the SE-type AS may be more involved in drought response of soybean roots. These results further indicate that AS may play an important role in regulating the drought response in soybean roots. 

### 3.5. Comparative Analysis of the Biological Functions of All DSGs Regulated at AS and Transcription Levels 

**Figure 5:**
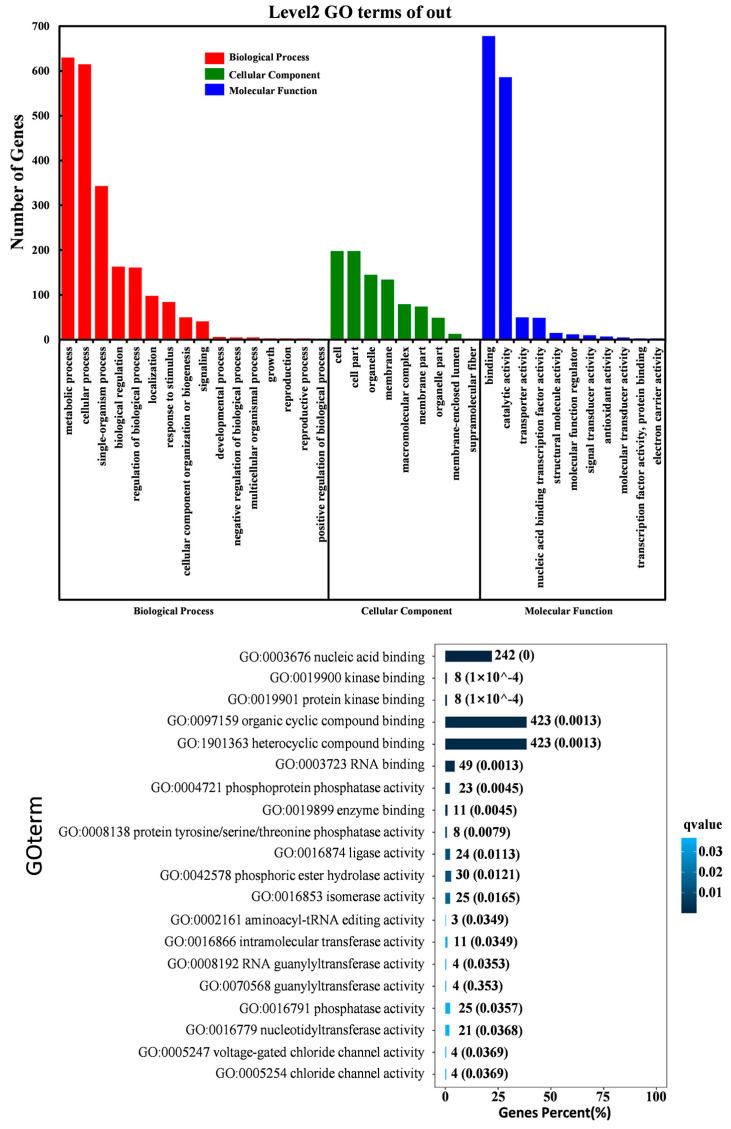
Representation of the Gene Ontology (GO) classification for biological process, molecular function, and cellular component. (**A**) GO terms assigned to DSGs under various drought conditions (false discovery rate (FDR) value < 0.05). (**B**) The top 20 GO enrichment terms (molecular function) of DSGs under various drought conditions. Notes: the blue bar chart represents the gene percentage (%) of the GO terms.

To investigate the biological functions that changed in response to drought and AS modulation, we performed Gene Ontology (GO) enrichment analysis was performed for all DSGs. As shown in Figure 5A, many genes involved in metabolic and cellular biological processes, as well as in the binding and catalytic activity involved in molecular function, were subject to AS regulation. Of particular interest were the many DSGs enriched in the following GO terms: nucleic acid binding, organic cyclic compound binding, and heterocyclic compound binding, suggesting that the AS regulation of binding activity had vital roles in the drought response of soybean root (Figure 5B). In addition, we found that the biological process related to the substance metabolic process and the primary metabolic process were significantly overrepresented (Figure S5). Those enriched biological processes, metabolic pathways, and biochemical activities provided an overview of the possible regulation of drought response in soybean roots through AS. 

### 3.6. Comparative Analysis of the Selected Differentially Expressed and Alternatively Spliced Genes 

**Figure 6:**
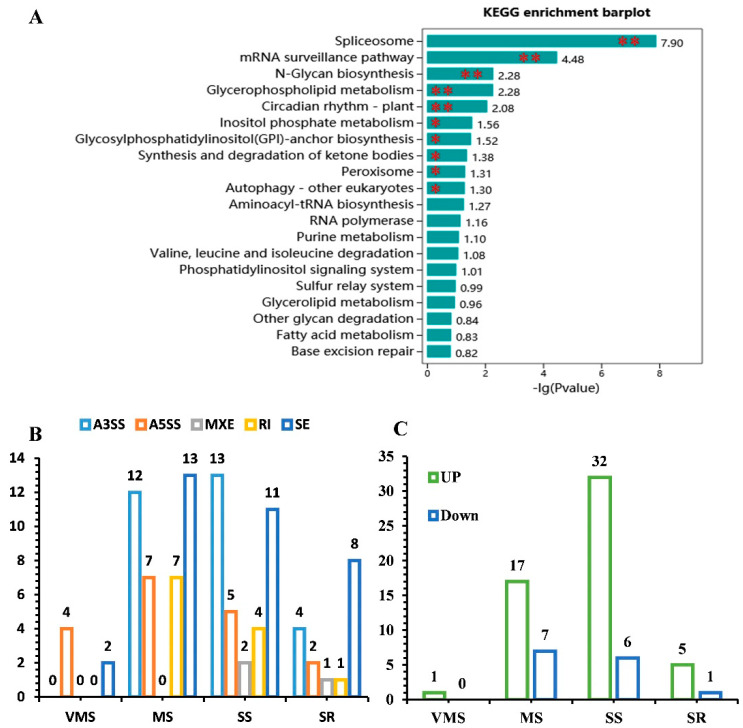
Expression and AS analysis of splicing regulatory factors (SPF)-related genes in response to various drought treatments. (**A**) The 20 most enriched Kyoto Encyclopedia of Genes and Genomes (KEGG) pathways (*G. max*. L.) of DSGs. The −Log10 (*p* value) was used to indicate the ratio of the DSG number and the number of genes annotated in this pathway. The greater the −Log10 (*p* value), the greater the degree of enrichment. **, *p* < 0.01; *, *p* < 0.05. (**B**) The number of significantly differentially spliced SPF-related genes identified under various drought retreatments. (**C**) The number of significantly differentially expressed SPF-related genes identified under various drought retreatments.

Splicing regulatory factors (SPFs), such as Ser/Arg-rich proteins, pre-mRNA splicing factors, and heterogeneous nuclear ribonucleoproteins (hnRNPs), are not only responsive to environmental stresses but are also alternatively spliced themselves under various environmental stresses [12,26,39,40]. KEGG pathway analysis was applied to evaluate whether such genes and pathways were enriched in the DSGs. We found that the spliceosome pathway (*p* value < 4.12 × 10^−10^) and mRNA surveillance pathway (*p* value < 3.28 × 10^−5^) were enriched in the DSGs (Figure 6A). These results indicate that the SPF-related genes in soybean also responded to drought stress and were alternatively spliced under drought conditions. 

In the present study, a total of 56 SPFs-related genes were also found to be significantly differentially spliced under various drought treatments. Among them, A3SS and SE types comprised the majority of AS types (Figure 6B). In addition, the number of upregulated SPF-related genes was greater than that of the downregulated genes (Figure 6C). The response of 49 SPF-related genes to various drought treatments was significantly different. Most SPF-related genes were upregulated in response to the MS and SS treatments compared with the VMS and SR treatments (Figure S6). These results indicate that the genes encoding the splicing factors were severely spliced in response to the drought stress of soybean roots. 

**Figure 7:**
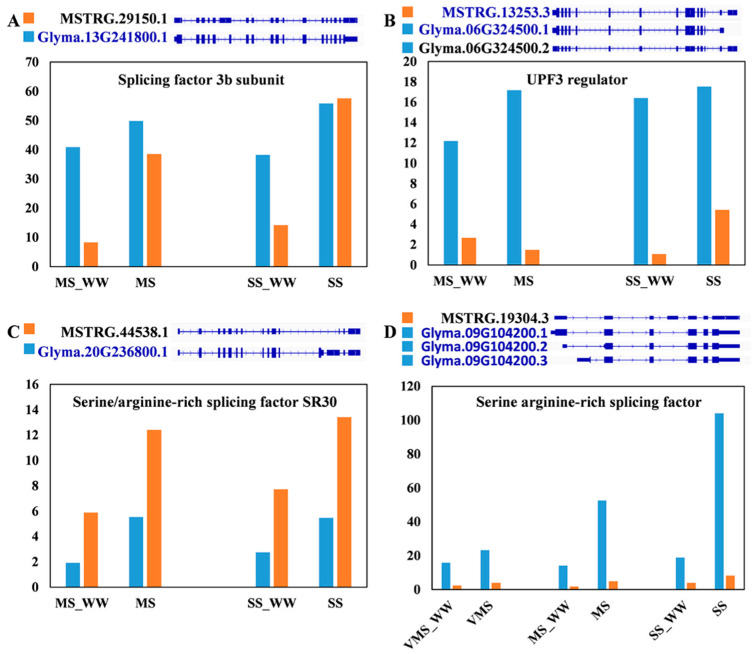
Visualization of representative transcript isoforms of four SPF-related genes to indicate expression changes under various drought stresses. MSTRG indicates the novel transcripts in this study. (**A**) Splicing factor 3b subunit (Glyma.13G241800) switched between MSTRG.29150.1 and Glyma.13G241800.1 under MS and SS conditions. Novel transcripts are illustrated as MXE events. (**B**) UPF3 regulator (Glyma.06G324500) switched between MSTRG.13253.3 and Glyma.06G324500 under MS and SS conditions. Novel transcripts are illustrated as A3SS events. (**C**) Serine arginine-rich splicing factor (Glyma.09G104200) switched between MSTRG.19304.3 and Glyma.09G104200.1, Glyma.09G104200.2, and Glyma.09G104200.3 under VMS, MS, and SS conditions, respectively. Novel transcripts are illustrated as A5SS events. (**D**) Serine/arginine-rich splicing factor SR30 (Glyma.20G236800) switched between MSTRG.44538.1 and Glyma.20G236800.1 under MS and SS conditions. Novel transcripts are illustrated as MXE events. The color of the functional gene ID is blue, while the color of nonfunctional gene ID is black.

To further reveal how the different stress-responsive transcripts responded to the various drought stresses, we investigated the expression patterns of four SPFs genes. As indicated in Figure 7A, the MS and SS treatments slightly induced the expression of Glyma.13G241800 (encoding the splicing factor 3b subunit), but greatly induced the expression level of a novel transcript (named MSTRG.29150.1). Conversely, the expression pattern of the novel transcript (named MSTRG.13253.3) of Glyma.06G324500 gene was downregulated under the MS condition but upregulated under the SS treatment (Figure 7B). In addition, the expression level of MSTRG.44538.1 was significantly higher than that of Glyma.20G236800 (Figure 7C). On the contrary, the expression level of MSTRG.19304.3 was significantly lower than that of Glyma.09G104200 (Figure 7D). These results indicate that different transcripts responded differently to drought and therefore may have different roles in regulating soybean response to drought. 

## 4. Discussion

### 4.1. Many AS Events Occurred in Soybean Roots under Various Drought Conditions

Abiotic stress-regulated AS events have not been systematically analyzed and reported at the whole transcriptome level in soybean, although AS has been studied in other plants and implicated in regulating plant stress responses [9,12,13,14,15]. In the present study, 385, 989, 1429, and 465 drought stress-responsive AS events were identified in soybean roots under VMS, MS, SS, and SR conditions, respectively, using whole transcriptome RNA-seq data. More AS events occurred under the MS and SS treatments than under the VMS and SR treatments, indicating that the number of AS events increased under the severe drought condition and decreased after re-watering. Therefore, our findings not only revealed a large number of AS events in soybean roots in response to various drought treatments, but also support the important roles of AS events in regulating soybean response to drought stress. 

### 4.2. A3SS and SE Were the Main AS Types in Soybean Roots in Response to Drought Stress

Although all five AS types were identified in the present study, SE and A3SS were the most abundant AS types and represented 67.1–69.0% of the total AS events under all the drought stress conditions (Figure S2). Further investigation showed that those two AS types accounted for 63.7–68.8% of all the drought stress-responsive AS events (Figure 2). Additionally, an approximately equal number of the observed AS events was found between A5SS and RI types under all conditions, but the number of A5SS type (85-294) was more than that of RI type (23-127) in all the stress-responsive AS events (Figure 2). On the contrary, RI was the most abundant AS event in different developmental stages of soybean [41,42,43]. RI was the major AS type that responded to drought, heat, and their combination in wheat and to nitrogen in maize [15,44]. The difference in dominant AS types between different developmental stages in soybean and between different plant species may be related to the regulation of SPF-related genes (both gene expression level and splicing events). Further work is needed to understand the observed difference.

### 4.3. A Number of Key GO Terms and Pathways Were Enriched in DSGs

DEGs were reported in a previous study and the significantly enriched KEGG pathways were related to the biosynthesis of secondary metabolites and phenylpropanoid biosynthesis, as well as metabolic pathways [35]. In the present study, the most enriched pathways in the DSGs were related to splicing and mRNA surveillance (Figure 6). In total, 85 (4%) DSGs were related to the spliceosome and mRNA surveillance pathways. The spliceosome processes pre-RNA to mature mRNA and is extensively involved in the splicing process [20,21]. Recently, it was reported that the modification of key spliceosome components is consistent with the transcriptional and proteome changes under drought stress in *Arabidopsis* [45]. In the present study, RNA-binding proteins (Glyma.03G214400, Glyma.05G003400) were also identified to be regulated at the splicing level, although a detailed composition of the spliceosome is yet to be characterized in soybean under drought stress. Therefore, it appears that specific spliceosome changes also occur in soybean roots under drought stress. 

The mRNA surveillance pathway is involved in the quality control of the accuracy of gene expression, as it detects and degrades abnormal mRNAs. Nonsense-mediated decay (NMD) is a major pathway of mRNA surveillance, which eliminates mRNAs containing premature translation termination codons (PTCs) [46]. It was reported that at least 17.4% of all multi-exon, protein-coding genes produce splicing variants that are targeted by NMD in *Arabidopsis*. UP FRAMESHIFT1 (UPF3), an NMD factor, could result in the de-capping and rapid exonucleolytic digestion of the mRNA [47]. UPF3 expression is feedback-regulated at multiple levels, and its balanced expression is essential for coping with salt stress in *Arabidopsis* [48]. Furthermore, the AS-coupled NMD was modulated by NaCl stress in *Arabidopsis* [47]. In our present study, one gene (Glyma.06G324500) encoding UPF3 regulator of nonsense transcripts homolog was found to be regulated by drought stress and AS (Figure 7D). Therefore, the mRNA surveillance pathway may also be involved in regulating soybean response to drought stress.

Plant splicing regulatory factors are known to be essential in regulating the AS of drought-related genes [12,26,39,40]. The interactions between Ser/Arg-rich protein and pre-mRNA are essential to constitutive and alternative splicing and have crucial contributions to maintaining cell and tissue homeostasis [12,49,50]. Interestingly, plant SPF-related genes themselves also undergo AS in a developmental and tissue-specific manner as well as in response to various hormones and abiotic stresses [10,12,51]. Overexpression of *BrSR45a* in *Arabidopsis* not only increased the abundance of the drought stress-inducible genes but also influenced the splicing patterns of target genes [52]. The transcript abundance and the ratio of exon-skipped transcripts of *AtSR45a* were promoted by heat and drought stress in *Arabidopsis* [53]. Notably, a number of genes encoding the Ser/Arg-rich proteins involved in pre-mRNA splicing were found to generate AS events in response to drought in the present study (Figure 6), for example, Glyma.16G121300, a homologue of SR45a. Likely the stress-dependent AS of SPF-related genes may also function in regulating soybean response to drought stress. 

The most enriched GO molecular functional terms in DSGs were related to nucleic acid binding, organic cyclic compound binding, and heterocyclic compound binding (Figure 5B). It was reported that nucleic acid binding and organic cyclic compound binding were significantly enriched in the OsMYBs regulatory network in drought response in rice [54]. The genes related to “organic cyclic compound binding and heterocyclic compound binding” were also over-represented under water deficit conditions in Chilean quinoa [55]. Therefore, in addition to drought-induced changes in gene expression levels, the drought-induced AS events may also play important roles in soybean root development. The enriched pathways may be conserved in different plant species in response to drought stress. 

### 4.4. The Complexity of the Steady State Transcriptome in Soybean Roots under Drought Conditions 

Our present and previous studies have demonstrated that the soybean transcriptome was not only regulated at the expression level, but also through the AS regulation. Previously, we showed that 6609 genes were differentially responsive to various drought conditions in soybean root [35]. Here, we revealed not only a large number of soybean genes that produced splicing variants that responded to drought conditions at transcript levels, but also a large number of genes that produced differentially spliced variants in response to different drought levels. Furthermore, 183 genes appeared to experience both changes in expression level and AS regulation in response to drought stresses. These results indicate the complexity of the transcriptome in soybean roots under drought conditions.

### 4.5. AS Has an Important Regulatory Role during Plant Development and Response to Abiotic Stress 

AS has been studied extensively in several plant species. The concept of deep learning and gene editing technology (Clusters of Regularly Interspaced Short Palindromic Repeats, CRISPR) have been used to identify AS events and gene function [52,56,57]. Moreover, differences in the recognition sequences of splice sites have been reported between plants and animals [26,58,59]. This might occur because plants have to endure unfavorable environments due to a sessile lifestyle. In particular, the conserved AS regulation model of key abiotic stress regulators among different plant species have been found, such as heat stress transcription factors (HsfA2) and dehydration-responsive element binding protein 2B (DREB2B) [17,18,60]. In addition, environmental stress regulates the changes of those AS regulators, and further regulates the AS and expression of other stress-related genes in plants, which ensures that plants can quickly respond and adapt to environmental changes [61,62,63,64,65].

With the development of high-throughput sequencing technology, more and more plant genome sequences and transcriptome data can provide technical support for the identification of species, tissues, developmental stages, and environmental specific regulation of AS, which help researchers to determine the dynamic changes of AS in different development stages and environmental conditions. Moreover, the research on the function and mechanism of AS in the process of abiotic stress can provide new strategies for enhancing stress tolerance of plants.

## 5. Conclusions

In summary, the present study revealed a large number of AS events that occurred in soybean roots in response to drought stress through analyzing the whole transcriptome RNA-seq data, providing a comprehensive view of AS in soybean roots under drought stress conditions. Therefore, in addition to the regular differential gene expression, our data suggest that drought-responsive AS events and/or their expression levels both act in a direct or indirect regulation mode to modulate soybean drought responses. Systemic approaches are further needed to dissect and understand soybean responses to drought stress and the roles of DSGs as well as DEGs in regulating such responses. The obtained knowledge will help develop future drought-resistant soybean cultivars.

## Supplementary Materials

The following are available online at https://www.mdpi.com/2073-4425/11/12/1520/s1: Figure S1: Quality and feature of the RNA-seq datasets used in this study. (A) Chromosome-wise read count distribution of all filtered reads in root samples. Reads were obtained throughout the soybean genome and distributed on different chromosomes using Williams 82 sequence as a reference. Raw reads were filtered using the criteria of MAPQ > 20 and insert size 100–1000 bp. (B) Normalized gene-body coverage of RNA-seq libraries generated from soybean root. For each library, the average coverage is shown at each relative position along the transcripts’ length. Figure S2: The number of different AS events in genes expressed in seedlings based on RNA-seq data compared with the annotated gene models under all drought conditions. Blue, exclusion; red, inclusion. Figure S3: Venn diagrams of the differentially spliced AS events derived from five AS types under various drought conditions: (A) SE type, (B) A3′SS type, (C) A5′SS type, (D) RI type, and (E) MXE type. Figure S4: AS profiles of significantly stress-responsive genes under various drought conditions. The bar charts show relative expression levels of alternatively spliced isoform that was well-watered (ww) (blue) and isoform stress (yellow) of AS genes under various drought conditions revealed by RNA-seq. The numbers in each bar indicate the expression percentages of different isoforms. Figure S5: The top 20 GO enrichment terms (biological process) of DEGs under all conditions. Notes: the blue bar chart represents the gene percent (%) of the GO terms. Figure S6: Clustering analysis of differentially expressed SPF-related genes under various drought treatments. The transcripts per million (TPM) values of expression levels of those genes were used. Upregulated (red) and downregulated (blue) SPF-related genes are presented by different color scales in the heatmap. Table S1: Raw data and clean data statistics of RNA sequencing. Table S2: The ID list of DSGs. Table S3: DSGs encoding novel transcripts under various drought conditions.

## Abbreviation

AS	alternative splicing
A5SS	alternative 5′ splicing site
A3SS	alternative 3′ splicing site
DAS	stress-responsive AS events
DREB2B	dehydration-responsive element binding protein 2B
DSGs	drought spliced genes
PTC	premature translation termination codon
FDR	false discovery rate calculated from *p*-value
GO	Gene Ontology
hnRNPs	heterogeneous nuclear ribonucleoproteins
IEPs	isoform expression percentage changes
IR	intron retention
KEGG	Kyoto Encyclopedia of Genes and Genomes
MXE	mutually exclusive exon
MS	mild drought stress
NMD	nonsense-mediated decay
rMATs	replicate multivariate analysis of transcript splicing
SE	skipped exon
SS	severe drought stress
SR	recovery after severe drought
SPFs	splicing regulatory factors
UPF3	UP FRAMESHIFT1
VMS	very mild drought stress
WW	well-watered control
