# Supplementary material for: Analysis of Whole Transcriptome RNA-seq Data Reveals Many Alternative Splicing Events in Soybean Roots under Drought Stress Conditions
Source: Genes (Basel). 2020 Dec 19;11(12):1520. doi: 10.3390/genes11121520 (PMC7765832; doi:10.3390/genes11121520)
Supplement: Supplementary file 1 [file genes-11-01520-s001.zip › Sup/Sup figure legend.docx]

**Figure S1**. **Quality and feature of the RNA-seq datasets was used in this study**. (**A**). Chromosome-wise read count distribution of all filtered reads in root samples. Reads were obtained throughout the soybean genome and distributed on different chromosomes using Williams 82 sequence as reference. Raw reads were filtered using the criteria of MAPQ>20 and insert size 100-1000 bp. (**B**). Normalized gene-body coverage of RNA-seq libraries generated from soybean root. For each library, the average coverage is shown at each relative position along the transcripts’ length.

**Figure S2**. The number of different AS events in genes expressed in seedlings based on RNA-seq data compared with the annotated gene models under all drought conditions. Blue, exclusion; red, inclusion.

**Figure S3**. Venn diagrams of the differentially spliced AS events derived from five AS types under various drought conditions. (**A**) SE type (**B**) A3'SS type (**C**) A5'SS type (**D**) RI type (**E**) MXE type

**Figure S4**. AS profiles of significantly stress-responsive genes under various drought conditions. The bar charts showed relative expression level of alternatively spliced isoform ww (blue) and isoform stress (yellow) of AS genes under various drought conditions revealed by RNA-seq. The numbers in each bars indicate the expression percentage of different isoforms.

**Figure S5**. The top 20 GO enrichment terms (biological process) of DEGs under all conditions. Notes: the blue bar chart represents the genes percent (%) of the GO terms.

**Figure S6**. Clustering analysis of differentially expressed SR-related genes under various drought treatments. The TPM value of those genes expression level was used. Up-regulated (red) and down-regulated (blue) SR-related genes were presented by different color scale in heatmap.

**Table S1.** Raw data and clean data statistics of RNA sequencing.

**Table S2. The** ID List of DSGs.

**Table S3.** DSGs that encoding novel transcripts under various drought conditions.
