# Supplementary material for: Analysis of Whole Transcriptome RNA-seq Data Reveals Many Alternative Splicing Events in Soybean Roots under Drought Stress Conditions
Source: Genes (Basel). 2020 Dec 19;11(12):1520. doi: 10.3390/genes11121520 (PMC7765832; doi:10.3390/genes11121520)
Supplement: Supplementary file 1 [file genes-11-01520-s001.zip › Sup/SUP-Ver 3-Song .pptx]

## Slide 1
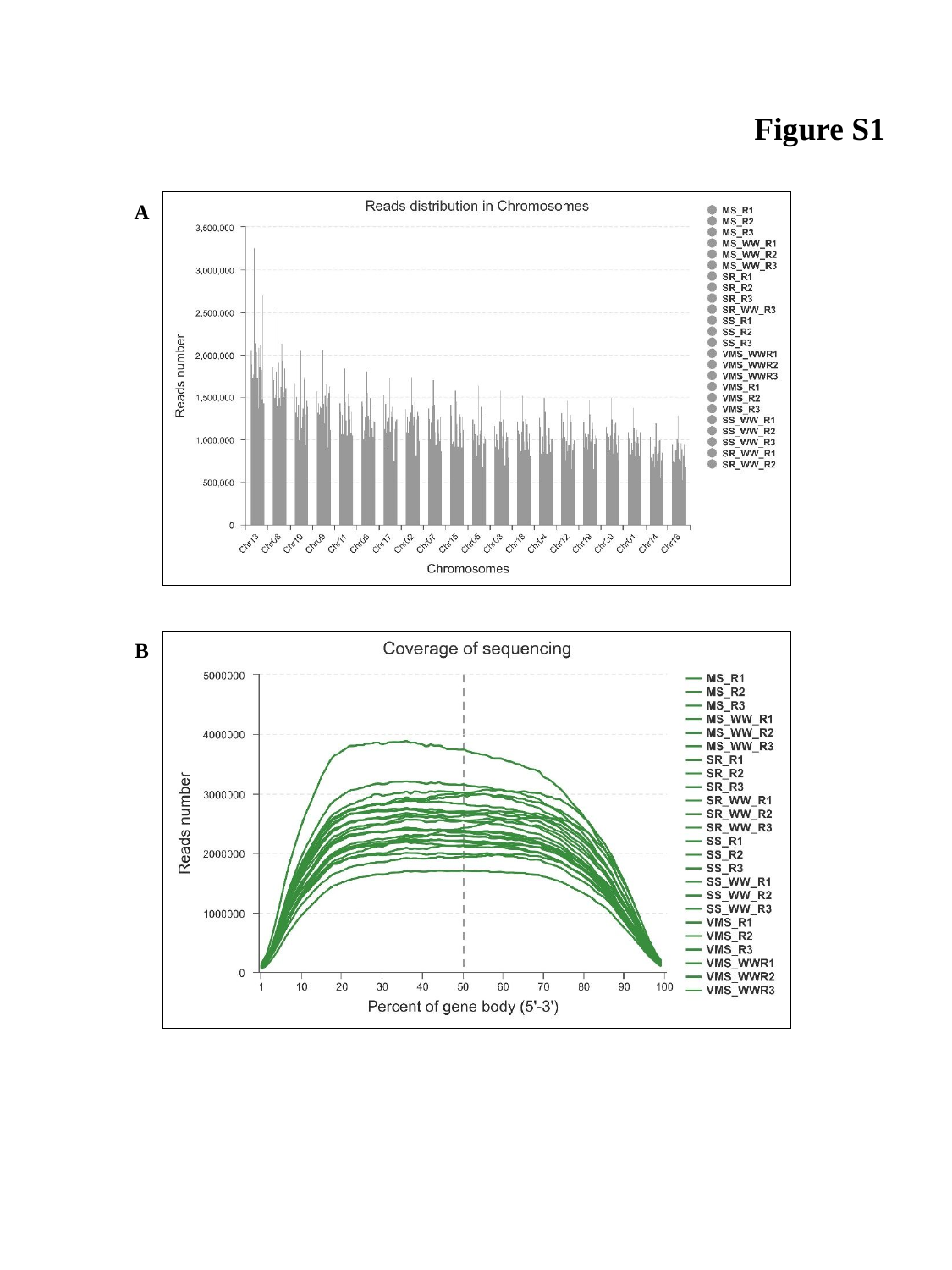

Figure S1
A
B

## Slide 2
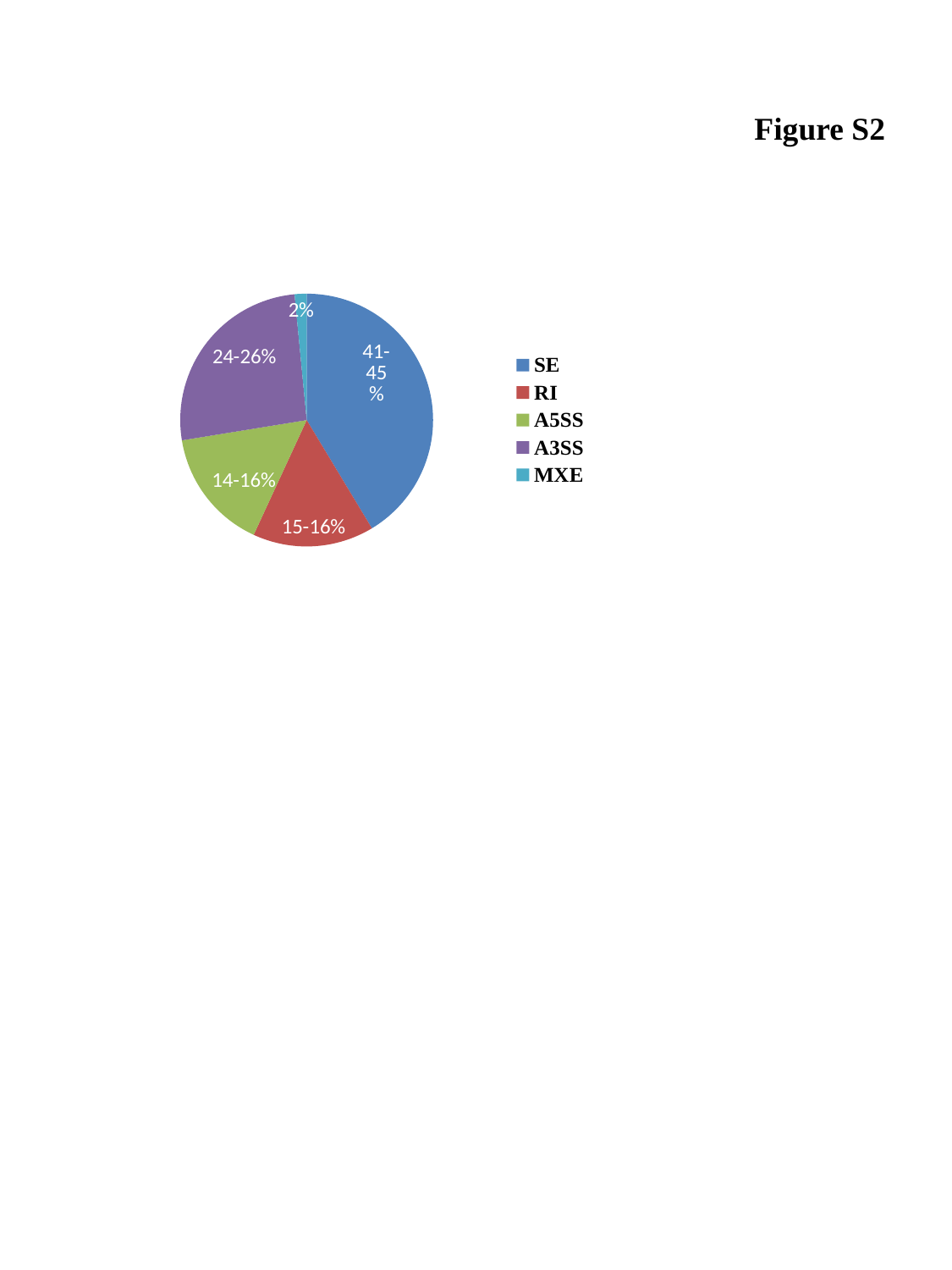

Figure S2
### Chart
| Category | VMS_WW |
|---|---|
| SE | 8260.666666666666 |
| RI | 3100.6666666666665 |
| A5SS | 3105.3333333333335 |
| A3SS | 5202.0 |
| MXE | 304.6666666666667 |

## Slide 3
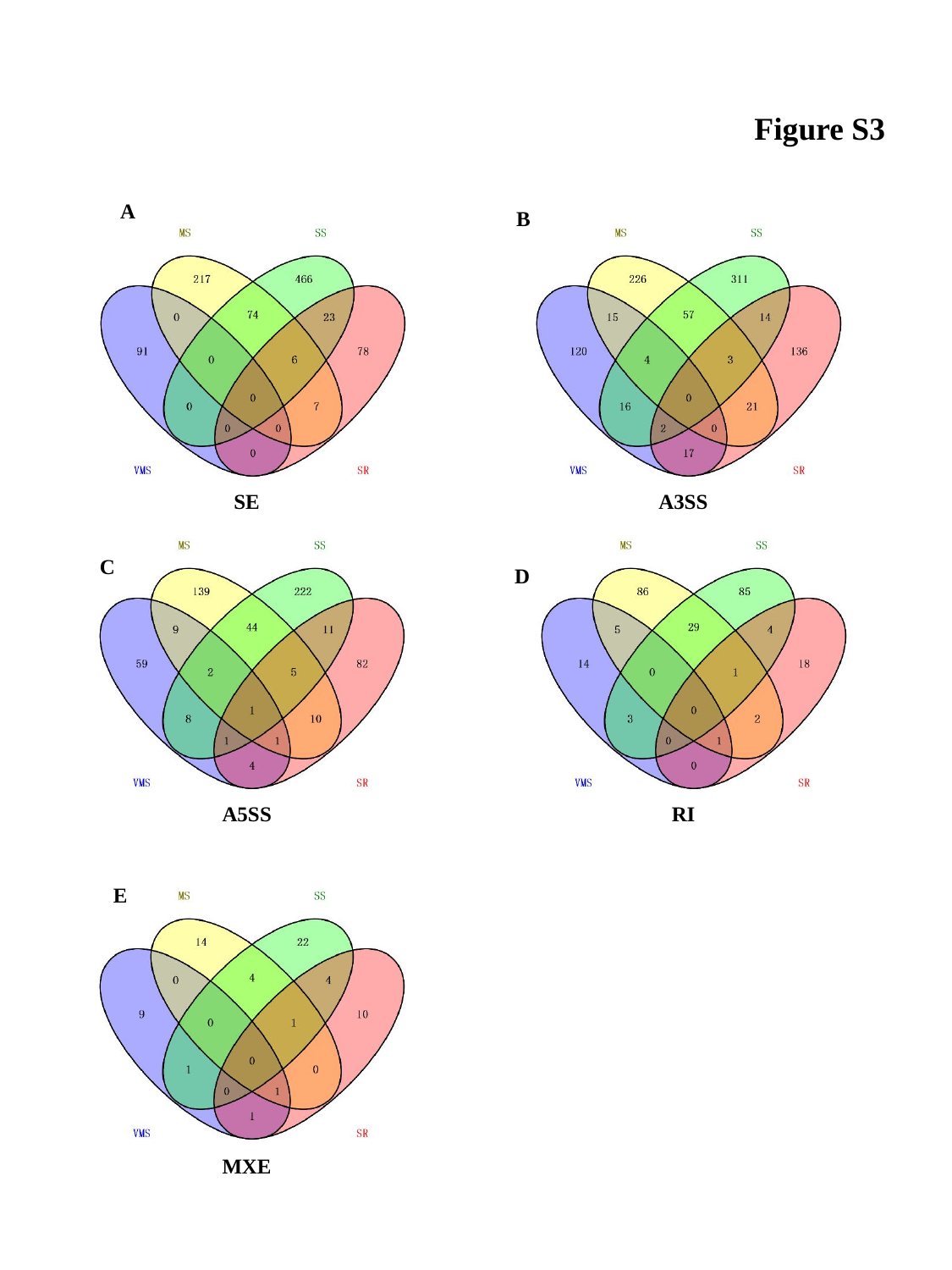

Figure S3
A
B
SE
A3SS
C
D
A5SS
RI
E
MXE

## Slide 4
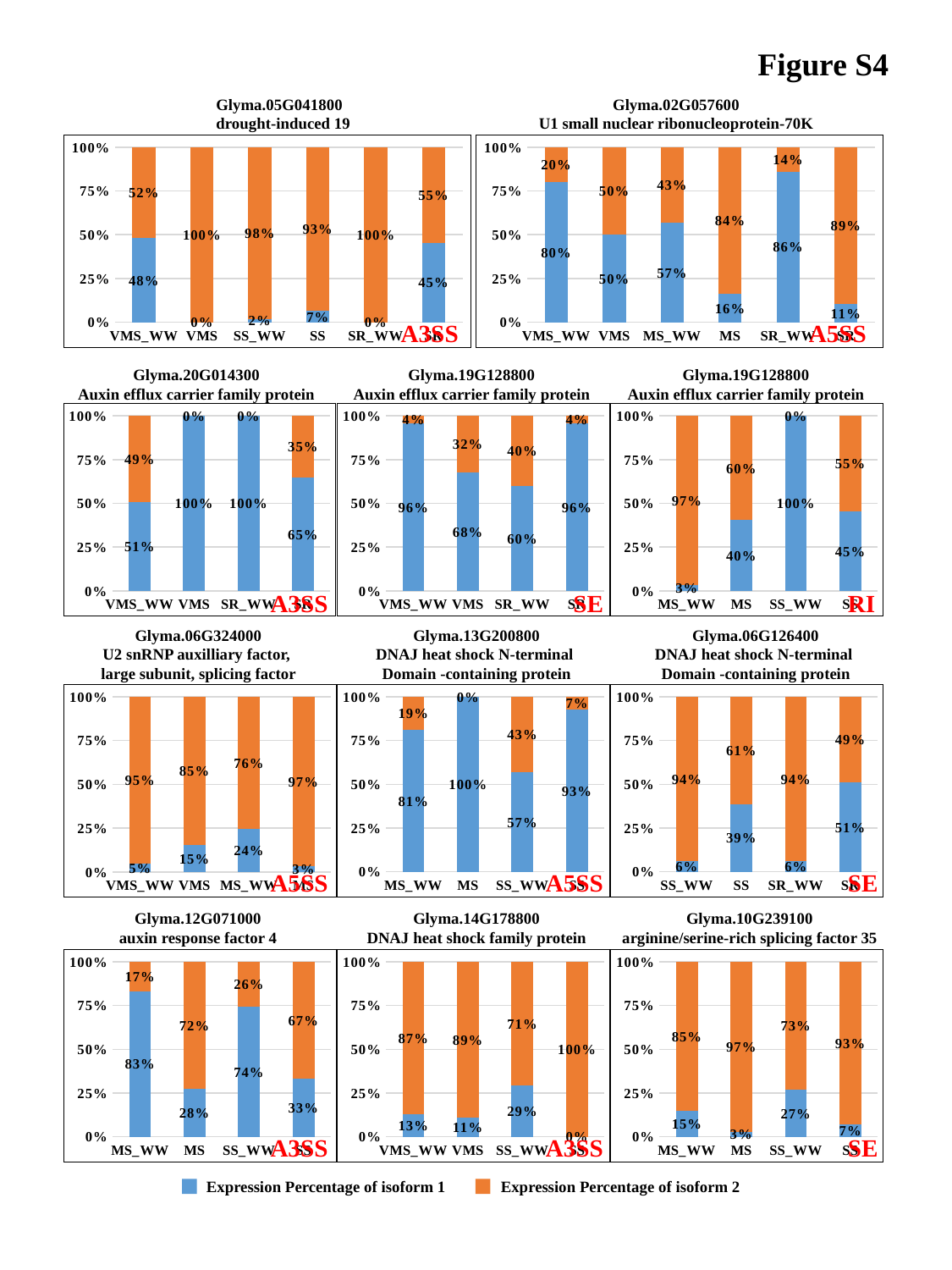

Figure S4
Glyma.05G041800
drought-induced 19
Glyma.02G057600
U1 small nuclear ribonucleoprotein-70K
### Chart
| Category | Iso 1 | Iso 2 |
|---|---|---|
| VMS_WW | 0.481333333333 | 0.518666666667 |
| VMS | 0.0 | 1.0 |
| SS_WW | 0.0196666666667 | 0.9803333333333 |
| SS | 0.0666666666667 | 0.9333333333333 |
| SR_WW | 0.0 | 1.0 |
| SR | 0.454 | 0.546 |
### Chart
| Category | Iso 1 | Iso 2 |
|---|---|---|
| VMS_WW | 0.8 | 0.19999999999999996 |
| VMS | 0.5 | 0.5 |
| MS_WW | 0.566666666667 | 0.43333333333299995 |
| MS | 0.160333333333 | 0.839666666667 |
| SR_WW | 0.860333333333 | 0.139666666667 |
| SR | 0.105333333333 | 0.894666666667 |A3SS
A5SS
Glyma.20G014300
Auxin efflux carrier family protein
Glyma.19G128800
Auxin efflux carrier family protein
Glyma.19G128800
Auxin efflux carrier family protein
### Chart
| Category | Iso 1 | Iso 2 |
|---|---|---|
| VMS_WW | 0.507 | 0.493 |
| VMS | 1.0 | 0.0 |
| SR_WW | 1.0 | 0.0 |
| SR | 0.646 | 0.354 |
### Chart
| Category | Iso 1 | Iso 2 |
|---|---|---|
| VMS_WW | 0.958333333333 | 0.04166666666700003 |
| VMS | 0.678 | 0.32199999999999995 |
| SR_WW | 0.600666666667 | 0.39933333333300003 |
| SR | 0.958333333333 | 0.04166666666700003 |
### Chart
| Category | Iso 1 | Iso 2 |
|---|---|---|
| MS_WW | 0.0333333333333 | 0.9666666666667 |
| MS | 0.404 | 0.596 |
| SS_WW | 1.0 | 0.0 |
| SS | 0.454 | 0.546 |A3SS
SE
RI
Glyma.06G324000
U2 snRNP auxilliary factor,
large subunit, splicing factor
Glyma.13G200800
DNAJ heat shock N-terminal
Domain -containing protein
Glyma.06G126400
DNAJ heat shock N-terminal
Domain -containing protein
### Chart
| Category | Iso 1 | Iso 2 |
|---|---|---|
| VMS_WW | 0.0476666666667 | 0.9523333333333 |
| VMS | 0.151666666667 | 0.848333333333 |
| MS_WW | 0.242666666667 | 0.757333333333 |
| MS | 0.0336666666667 | 0.9663333333333 |
### Chart
| Category | Iso 1 | Iso 2 |
|---|---|---|
| MS_WW | 0.81 | 0.18999999999999995 |
| MS | 1.0 | 0.0 |
| SS_WW | 0.569 | 0.43100000000000005 |
| SS | 0.926 | 0.07399999999999995 |
### Chart
| Category | Iso 1 | Iso 2 |
|---|---|---|
| SS_WW | 0.0623333333333 | 0.9376666666667 |
| SS | 0.387 | 0.613 |
| SR_WW | 0.0623333333333 | 0.9376666666667 |
| SR | 0.510666666667 | 0.489333333333 |A5SS
A5SS
SE
Glyma.12G071000
auxin response factor 4
Glyma.14G178800
DNAJ heat shock family protein
Glyma.10G239100
arginine/serine-rich splicing factor 35
### Chart
| Category | Iso 1 | Iso 2 |
|---|---|---|
| MS_WW | 0.832333333333 | 0.16766666666700003 |
| MS | 0.275666666667 | 0.724333333333 |
| SS_WW | 0.741 | 0.259 |
| SS | 0.333333333333 | 0.666666666667 |
### Chart
| Category | Iso 1 | Iso 2 |
|---|---|---|
| VMS_WW | 0.127333333333 | 0.872666666667 |
| VMS | 0.110666666667 | 0.889333333333 |
| SS_WW | 0.294 | 0.706 |
| SS | 0.0 | 1.0 |
### Chart
| Category | Iso 1 | Iso 2 |
|---|---|---|
| MS_WW | 0.147666666667 | 0.852333333333 |
| MS | 0.0296666666667 | 0.9703333333333 |
| SS_WW | 0.269333333333 | 0.730666666667 |
| SS | 0.073 | 0.927 |A3SS
A3SS
SE
Expression Percentage of isoform 1
Expression Percentage of isoform 2

## Slide 5
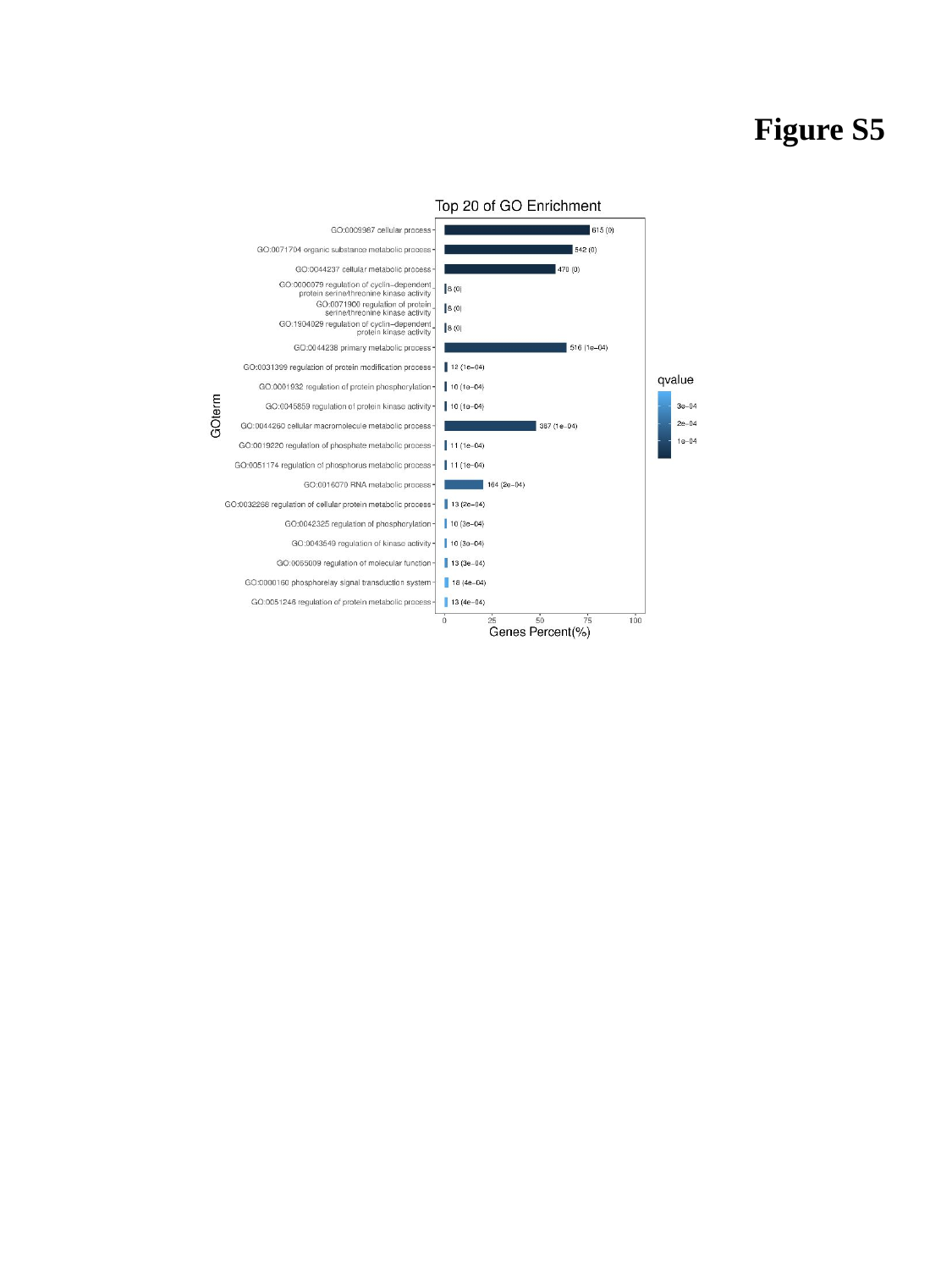

Figure S5

## Slide 6
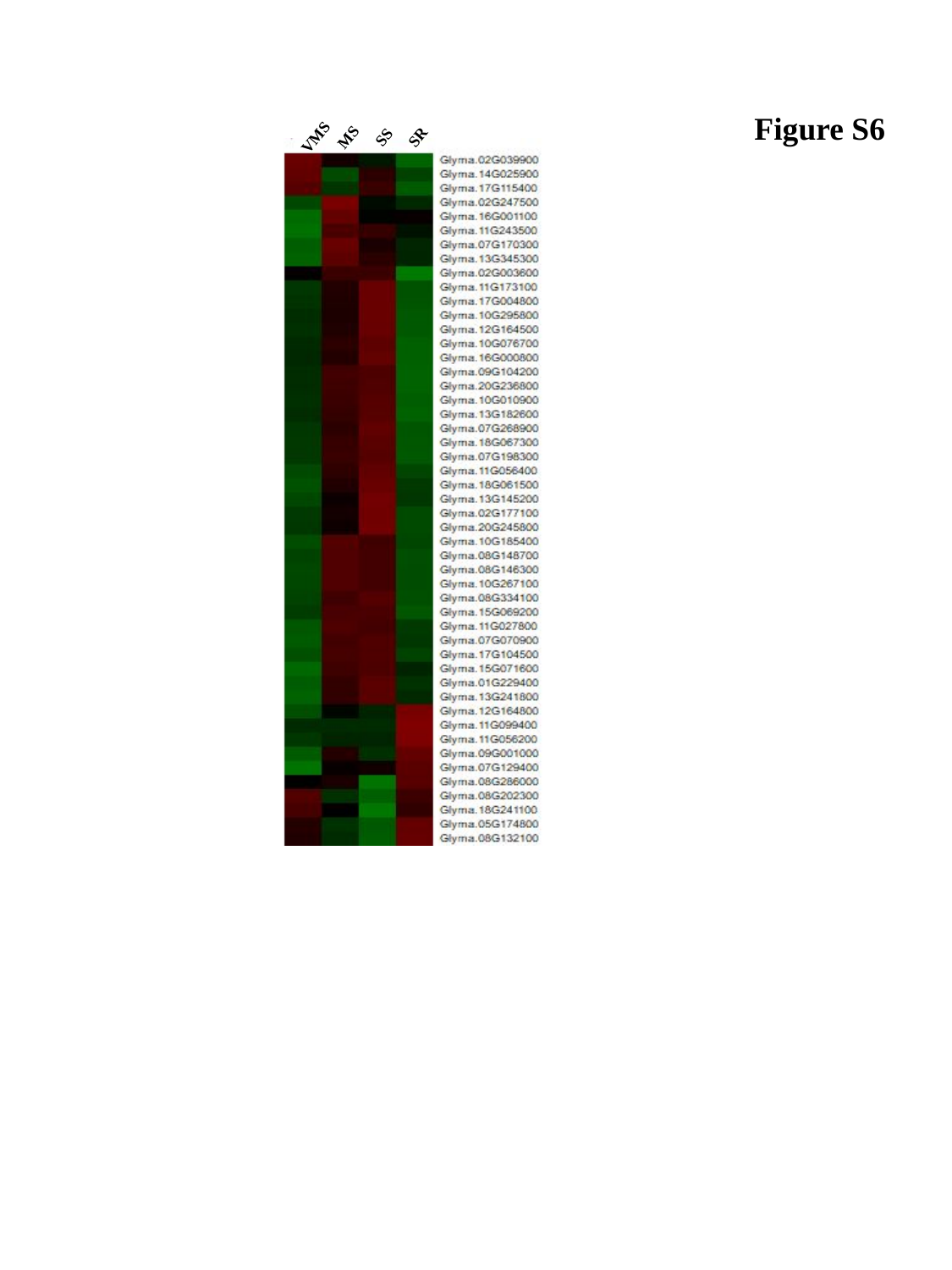

Figure S6
VMS
MS
SS
SR
